# Does Parental Mind‐Mindedness Account for Cross‐Cultural Differences in Preschoolers’ Theory of Mind?

**DOI:** 10.1111/cdev.12746

**Published:** 2017-02-03

**Authors:** Claire Hughes, Rory T. Devine, Zhenlin Wang

**Affiliations:** ^1^ University of Cambridge; ^2^ The Education University of Hong Kong

## Abstract

This study of 241 parent–child dyads from the United Kingdom (*N *=* *120, *M*_age_ = 3.92, *SD* = 0.53) and Hong Kong (*N *=* *121, *M*_age_ = 3.99, *SD* = 0.50) breaks new ground by adopting a cross‐cultural approach to investigate children's theory of mind and parental mind‐mindedness. Relative to the Hong Kong sample, U.K. children showed superior theory‐of‐mind performance and U.K. parents showed greater levels of mind‐mindedness. Within both cultures parental mind‐mindedness was correlated with theory of mind. Mind‐mindedness also accounted for cultural differences in preschoolers’ theory of mind. We argue that children's family environments might shed light on how culture shapes children's theory of mind.

Early accounts of how children acquire an understanding of others’ minds (or a “theory of mind”) adopted a nativist and modular perspective that highlighted the universality of developmental pathways and assumed minimal environmental influence (e.g., Carruthers, 2013; Scholl & Leslie, 2001). Challenging this view, several strands of recent research have highlighted the importance of early social experiences in the development of children's theory of mind (Hughes & Devine, 2015). For example, Heyes and Frith (2014) have argued that learning to read minds is “a slow effortful process in which a novice develops an important, culture‐specific skill through expert tuition” (p. 1357). Supporting this argument, the rate at which milestones in theory of mind are acquired (indexed by children's performance on the false belief task) can vary significantly across cultures. Although some studies emphasize similarities across cultures (e.g., Callaghan et al., 2005; Vinden, 1999), a growing number highlight marked contrasts (e.g., Lecce & Hughes, 2010; Hughes et al., 2014; Liu, Wellman, Tardif, & Sabbagh, 2008).

The precise processes underpinning cross‐cultural differences in children's understanding of mind have yet to be identified. One interpretation is that there are genuine cross‐cultural differences in children's theory of mind that are attributable to contrasts in children's social–cultural environments (e.g., parental behaviors). An alternative interpretation of these findings is that apparent cultural differences in task performance might reflect methodological confounds. Our study aimed to shed new light on cultural differences in preschool children's false belief understanding by assessing the impact of a key aspect of children's social environments and by addressing methodological concerns in this field.

From a sociocultural perspective, differences across cultures might reflect contrasts in the relative salience of mental states within specific cultures (e.g., Lillard, 1998; Taumoepeau, 2015). In other words, delayed false belief understanding is to be expected in cultures in which mental states are not appropriate objects for conjecture. This approach has led to a widespread distinction between “East” and “West,” or more specifically, cultures that, historically, have inherited the philosophical traditions of Ancient China or Ancient Greece (Nisbett & Masuda, 2003). However, meta‐analytic findings underscore the importance of looking beyond this simple East–West contrast. Specifically, drawing on data from single culture studies, Liu et al. (2008) have found that, relative to their North American peers, delayed false belief understanding is characteristic of Chinese children living in Hong Kong but not in mainland China.

The delayed acquisition of a theory of mind among young children living in Hong Kong (relative to both children in mainland China and children from Canada and North America) is puzzling. In particular, unlike English, both Mandarin and Cantonese are lexically explicit in referring to false (as opposed to true) beliefs. Moreover, compared with children living in mainland China, children living in Hong Kong are more likely to have siblings and to be bilingual: two factors that are believed to facilitate theory‐of‐mind development (Adesope, Lavin, Thompson, & Ungerleider, 2010). One possible explanation for the apparent delay in theory‐of‐mind development among Hong Kong children hinges on contrasts in parenting styles. For example, in a survey‐based study of over 2,000 adults living in China, Taiwan, and Hong Kong, Berndt, Cheung, Lau, Hau, and Lew (1993) found that adults in Hong Kong perceived their parents as having showed less warmth and more control than parents in China or Taiwan. Lai, Zhang, and Wang (2000) reported similar findings based on a parental self‐report Q‐sort task comparing mothers from Hong Kong and Beijing. To date, however, no study has directly compared Hong Kong preschool children's false‐belief understanding with that of children living in the West. A first preliminary aim of this study was therefore to address this gap in the literature using a comparison group that was recruited from the United Kingdom. Here, it is worth noting that the similarities in educational systems between these two countries (reflecting a century of British control in Hong Kong) enabled us to focus on familial factors that might account for the predicted mean group difference in false‐belief task performance.

Interestingly, with a few exceptions (e.g., Lecce & Hughes, 2010; Sabbagh, Xu, Carlson, Moses, & Lee, 2006), cross‐cultural comparisons have typically overlooked family factors that might influence children's false belief understanding. Factors such as socioeconomic status (SES) and family size each show positive associations (of similar strength, *r* ≈ .20) with variation in false belief performance (Devine & Hughes, 2018). The latter of these associations is, at first glance, counterintuitive because family size is typically inversely related to both language and general cognitive ability, leading theorists to posit models of “resource dilution” (e.g., Downey, 2001). However, as detailed observational studies make clear, the presence of siblings provides young children with multiple opportunities for learning about others’ minds through pretend play, conflict, and observing family interactions (e.g., Hughes & Devine, 2015). In the present study, which focused on how *parents* may facilitate children's emerging understanding of mind, measures of SES and family size were included as control variables.

A key question for the present study was whether, in each site, within‐country variation in children's false belief performance would be associated with variation in parental “mind‐mindedness,” that is, parents’ proclivity to view their children as mental agents. Meins, Fernyhough, Russell, and Clark‐Carter (1998) introduced the concept of parental mind‐mindedness to the field in an effort to examine how parents shape the development of children's theory of mind. Online or “interactional” measures of mind‐mindedness are derived from recording the number of “appropriate” versus “nonattuned” mind‐related comments that parents make about their children during play interactions (Meins et al., 2012). Offline or “representational” measures of mind‐mindedness are derived from parents’ descriptions of their children that are then coded for the relative frequency of reference to either mental or nonmental (i.e., behavioral, physical, general) attributes (Meins et al., 1998). Offline measures are more suitable for studying preschool children than online measures. Online measures of appropriate mind‐related comments in infancy are moderately correlated with later use of mental descriptions in the offline task (Meins et al., 2003), and both indices of parental mind‐mindedness show moderate associations with individual differences in children's false belief understanding (e.g., Laranjo, Bernier, Meins, & Carlson, 2014; Lundy, 2013; Meins et al., 1998).

To our knowledge, this is the first cross‐cultural study to examine parental mind‐mindedness. There are at least two reasons why one might expect between‐culture contrasts in parental mind‐mindedness. First, evidence from several separate studies (e.g., Meins, Fernyhough, Fradley, & Tuckey, 2001; Meins, Fernyhough, Arnott, Leekam, & DeRosnay, 2013; Meins et al., 2002) indicates that the concept of parental mind‐mindedness is related to (but distinct from) that of parental sensitivity, which is typically defined as parents’ ability to “tune in” and respond to the needs of their children. In turn, numerous studies (e.g., Bornstein et al., 1992; Bornstein, Putnick, Cote, Haynes, & Suwalsky, 2015; Posada et al., 2016) have shown that sensitive parental behavior and parent–infant contingent talk are culturally universal but vary in overall amount and quality across different cultural settings. A second reason for expecting cross‐cultural contrasts in parental mind‐mindedness is that, as noted earlier, cultures differ in the degree to which mental states are viewed as objects worthy of conjecture (Lillard, 1998). Indeed parents’ propensity to use mental‐state language with their children differs according to their cultural background (e.g., Wang, Doan, & Song, 2010).

In addition to investigating between‐country contrasts in mean levels of parental mind‐mindedness, this study provided an opportunity to examine, for the first time, whether the association between parental mind‐mindedness and children's false belief understanding is culturally invariant. In their discussion of parenting across different Chinese cultures, Wang and Chang (2012) raise a point that deserves note in relation to the current study. Specifically, mean‐level differences across cultures in particular parenting behaviors might reflect the salience of those behaviors in a particular cultural setting and so do not necessarily signal differences in the developmental relations between particular parental behaviors and specific child outcomes. That is, cultural contrasts in mean levels of a specific parental attribute (such as mind‐mindedness) can co‐occur with culturally invariant patterns of association with specific child outcomes (such as false belief performance). In the absence of direct evidence for cultural contrasts in the correlates of parental mind‐mindedness, we applied the principle of parsimony to predict that its association with children's false belief performance would be culturally universal.

Turning to alternative methodological accounts of cross‐cultural contrasts, several points deserve note. First, evidence shows that individual differences in theory of mind are closely linked with child characteristics, such as age (Wellman, Cross, & Watson, 2001), language ability (Milligan, Astington, & Dack, 2007), and family size (e.g., Peterson, 2000). Given these associations, it is surprising that, with few exceptions (e.g., Hughes et al., 2014; Sabbagh et al., 2006), previous studies have not attempted to control statistically for important child characteristics, making group differences difficult to interpret. Second, the validity of applying false‐belief tasks (developed originally in Europe) in non‐European settings has been challenged (e.g., Lillard, 1998). Indeed, in Chinese cultures the choice of verb in the test question impacts upon children's performance. Specifically, Liu et al. (2008) found that using a “marked” belief verb (i.e., “think falsely”) makes the false‐belief task easier than when using a “neutral” belief verb. To ensure that any between‐group difference did not reflect this contrast in lexical ambiguity, we took several steps to remove these confounds (e.g., by choosing neutral verbs such as “look/zaau2” and “think/gok3 dak1”).

Third, it is now recognized that group differences can arise in cross‐cultural research for reasons unrelated to the underlying ability in question (Brown, 2006; Chen, 2008). Rather than reflecting a genuine difference in children's understanding of mind, group differences can appear if test items have different cultural meanings that interfere with responding (Byrne & Campbell, 1999). This “forks *versus* chopsticks” (Chen, 2008) account highlights the importance of testing that the data show measurement invariance across groups. Cross‐cultural studies in psychology have traditionally adopted analytic approaches (e.g., analysis of variance, *t* tests, chi‐square) that assume that test items function in a similar way for all participants regardless of group membership (e.g., Byrne & Campbell, 1999). Such analytic approaches conflate task‐specific variance, error variance, and performance on the underlying latent factor, and so might generate misleading results regarding any potential cross‐cultural differences. The assumption of measurement invariance (i.e., whether test items exhibit equivalent relations with underlying latent constructs in different groups) for all participants in a study can be tested using confirmatory factor analysis (CFA) and latent variable modeling (Brown, 2006). A latent variable analytic approach to cross‐cultural data analysis confers several benefits: (a) It is hypothesis driven and so enables researchers to specify measurement models in advance, (b) multiple groups CFA permits researchers to examine the stability of a measurement model across different groups, and (c) latent variable analyses use error‐free “true” scores that provide better estimates of group differences or associations between variables (Brown, 2006; Kline, 2011). In the only cross‐cultural theory‐of‐mind study to adopt this approach, Hughes et al. (2014) compared performance on a battery of false‐belief tasks in 5‐ and 6‐year‐old children in the United Kingdom, Italy, and Japan, and found only partial measurement invariance highlighting the need for measurement invariance to be tested rather than assumed. For example, Italian children appeared unfairly disadvantaged by one test item that concerned a parent lying to her child underscoring the potential of test bias to distort findings.

In sum, the current study involved parent–child dyads from Hong Kong and the United Kingdom and examined cultural contrasts in parental mind‐mindedness and in children's false belief performance, associations between these two measures, and whether the expected group difference in children's false‐belief task performance could be explained by a parallel contrast in parental mind‐mindedness. In addition, we sought to address the methodological problems encountered in previous studies by ensuring there were no significant differences between our samples in age, gender, and ability; preparing “culturally fair” testing materials; and testing the measurement equivalence of the false‐belief task battery prior to performing group comparisons.

## Method

### Participants

The data for this study were collected between March and December 2014. The U.K. sample consisted of 120 (61 males) parent–child dyads recruited from local nurseries, playgroups, parent mailing lists, libraries, and shopping centers in Cambridge, United Kingdom. We recruited 127 parent–child dyads. Seven children were excluded from the final sample due to noncompliance/noncompletion of tasks (*N *=* *5) or failure of control questions on the false belief task (*N *=* *2). The Hong Kong sample consisted of 121 (61 males) parent–child dyads recruited from local kindergartens in New Territories. Initially 131 parent–child dyads were recruited. Nine children were excluded from the final sample due to failure of control questions on the false belief task, and another one was excluded due to a later parental report of developmental delay. To minimize the potential effects of confounding variables, we set out to ensure that there were no differences between the two samples in gender composition and age.

The two samples were matched in gender composition, χ^2^(1) = .004, *p* = .95, and age, *M*_UK_ = 3.92, *SD* = 0.53, range = 3.00–4.95 years; *M*_HK_ = 3.99, *SD* = 0.50, range = 3.09–4.99 years, *t*(239) = 1.08, *p* = .28. In addition, there were no significant differences between the two groups in the total number of siblings, *M*_UK_ = 0.92, *SD* = 0.89; *M*_HK_ = 0.79, *SD* = 0.75, *t*(239) = 1.15, *p* = .25, or number of child‐aged siblings, *M*_UK_ = 0.68, *SD* = 0.61; *M*_HK_ = 0.61, *SD* = 0.64, *t*(239) = 0.89, *p* = .37, performance on tests of nonverbal ability, *M*_UK_ = 16.85, *SD* = 8.21; *M*_HK_ = 18.21, *SD* = 7.62, *t*(239) = 1.33, *p* = .18, and expressive language ability, *M*_UK_ = 15.12, *SD* = 7.43; *M*_HK_ = 14.43, *SD* = 6.88, *t*(239) = .75, *p* = .46. There were significant differences in levels of parental education. Parents in the United Kingdom (*N *=* *98) were more likely to have a degree level education than parents in Hong Kong (*N *=* *42), χ^2^(1) = 53.76, *p* < .001. This reflects local trends in higher level education. Approximately 20% of adults in Hong Kong and almost 55% of adults in Cambridge have a degree level education (Hong Kong Education Bureau, n.d.; Office of National Statistics, 2012). Children in Hong Kong spent significantly more hours per week at nursery, *M*_HK_ = 26.63 hr, *SD* = 11.97, than children in the United Kingdom, *M*_UK_ = 18.85 hr, *SD* = 11.02, *t*(239) = 5.25, *p* < .001. Given these differences in parental education and nursery attendance, we included these measures as covariates in our models.

### Procedure

All procedures were approved by the local university research ethics committees. Parents and children took part in a session lasting up to 90 min in an observation laboratory at each university. Children completed a battery of tasks designed to measure false belief understanding, verbal ability, and nonverbal ability. Individual child cognitive testing lasted approximately 30 min. The children in both sites completed additional structured parent–child play observations (not reported here). In an adjoining room, parents completed a short demographic questionnaire and an interview. The children completed the task battery in a fixed counterbalanced format with each false‐belief task being alternated with language and nonverbal ability measures. Children were provided with rest breaks and rewarded with stickers for completion of tasks. Parents were debriefed at the end of each session and provided with £15 or HK$50, and children received a small gift for taking part. Testing sessions followed a detailed manual to ensure that the procedures in each site were identical. The same stimuli and materials were used in both sites of the study. In the United Kingdom, all testing sessions were completed in English and in Hong Kong, all testing sessions were completed in Cantonese. All materials were prepared in English first and then translated into Cantonese by a panel of three English/Cantonese bilingual psychologists that included the third author, adopting a collaborative and iterative translation approach to ensure conceptual equivalence (Douglas & Craig, 2007).

### Measures

#### Children's False Belief Understanding

The children completed four tasks designed to measure individual differences in false belief understanding.

##### Change of location false‐belief task (Baron‐Cohen, Leslie, & Frith, 1985; Perner, Mauer, & Hildenbrand, 2011)

The children completed two separate change of location tasks. Both tasks were administered using specially prepared picture stimuli developed by the authors that involved two sets of characters (i.e., Su and Shaun, Andy and Sally). In both stories, the children were introduced to a story character who places an object in one location (e.g., a cupboard or a drawer) before going out to play. In the character's absence, another character enters the scene and places the object in a different location. Following these events, the children were then asked three forced‐choice questions to assess their memory for the events in the story (e.g., “Where is the book now?,” “Who put it there?,” “Where did Sally put the book in the beginning?”). If children failed any of these three initial questions, the experimenter reread the first part of the story. If the participant continued to fail any one of these items, testing was discontinued (*N *=* *2 in United Kingdom, *N *=* *9 in Hong Kong). Following these three questions, the story continued with the character returning to the scene. The experimenter then asked the false belief prediction question (e.g., “Where will Sally look for her book?”). In both the English and the Cantonese versions, we chose to use the neutral verb “look” in these questions (“zaau2” in Cantonese). Children scored 1 point for a correct response and 0 points for an incorrect response. Following this question, the experimenter showed a final image of the character searching for the object in the location he or she left it in. The children were then asked the false belief explanation question (e.g., “Why did Sally look for her book in the cupboard?”).

Children's explanations were coded using the scheme reported by Perner et al. (2011) with “correct” answers including both implicit (“it was in there earlier”) and explicit (“she thought it was in there”) explanations (1 point) and “incorrect” answers referring to information about the character's desires or irrelevant facts (0 points). Consistent with previous studies (e.g., Hughes et al., 2014), the scores from the prediction and explanation question were summed together so that children received a total possible score of 2 points for each task.

##### Unexpected contents false‐belief task (Gopnik & Astington, 1988)

For this task, the children were shown a box of plasters from Hong Kong, which had both English and Cantonese labeling and depicted a clear image of a plaster. This box contained some crayons. First, the children were asked what was in the box. Underscoring the fairness of these test materials, no child in either country failed this question. Following this, the children were then asked to look inside the box and tell the experimenter what was inside. After this the children were asked to return the crayons to the box and seal it up. The children were then asked three forced‐choice questions. First, children were asked the representational change question (i.e., “Before you looked inside, what did you think was inside the box?”). This was followed by a reality control question (i.e., “What's in the box really?”). Finally children were asked the false belief question (i.e., “Your mummy hasn't seen what's inside this box. If she sees this box all closed up, what will she think is inside it?”). In both the English and the Cantonese version, we chose to use the neutral verb “think” in these questions (“gok3 dak1” in Cantonese). To be credited with passing either question, children had to pass the reality control question. Responses were summed to give a total possible score of 2 points.

##### Unexpected identity false‐belief task (Hughes, 1998; Taylor, Cartwright, & Bowden, 1991)

In this task, the experimenter introduced a wordless pop‐up picture book (Moerbeek, 1994). On each page the experimenter pointed to a picture that appeared to be an eye peeping through the page and asked the child “What's that?” Upon turning the page, the picture was revealed to be an animal's eye. After five consecutive trials, the experimenter reached the penultimate page and pointed to the picture that appeared to be an eye and asked “What's that?” Upon turning the page, the experimenter revealed that the picture was in fact a spot on a snake's back. The experimenter then turned back to the penultimate page and asked three forced‐choice questions. First, children were asked a representational change question (i.e., “What did you think it was before we turned the page?”). Next children were asked a reality control question (i.e., “What is it really?”). Finally, children were asked a false belief question (i.e., “Your mummy hasn't seen this book before. If she sees this page what will she think it is?”). Again, neutral verbs, either “think” or “gok3 dak1,” were used in these questions. To pass either question, children had to pass the reality control question. Responses were summed together to give a total possible score of 2.

#### Children's Language Ability

Although measures of receptive vocabulary (e.g., Peabody Picture Vocabulary Test) are widely used in research on children's theory of mind, the absence of information about the relative difficulty of particular words in different languages means that these measures are not appropriate for use across different cultures. Moreover, meta‐analytic data show that there are no differences in the strength of correlation between different measures of language ability (i.e., measures of semantics vs. syntax) and individual differences in false belief understanding (Milligan et al., 2007). To provide a culturally fair measure of language ability the children in both sites completed the Bus Story Test (Renfrew, 1997; Stokes & Wong, 1996). This task was available in both English and Cantonese. The Bus Story information score provided us with a culturally fair measure of children's language ability. Rather than scoring children's use of specific vocabulary, linguistic complexity, syntax, or sentence length, the information score in both the English and Cantonese version represents the number of story elements in the children's narratives. The information score captures both expressive and receptive language abilities in that children must comprehend the narrative and then retell it (Kovas et al., 2005). The information score is strongly correlated with measures of receptive vocabulary, and like measures of receptive vocabulary, it exhibits moderate correlations with children's false belief task performance (e.g., Adams & Gathercole, 1996; Cutting & Dunn, 1999). In both sites, the experimenter read aloud a short narrative accompanied by a set of pictures, and the children were asked to retell the story using the picture stimuli as prompts. The children's narratives were digitally recorded and transcribed verbatim. These stories were scored for total information (i.e., 1 point for each story element in their narrative).

#### Children's Nonverbal Ability

To provide an index of nonverbal ability, the children in both sites completed the *object assembly* task from the Wechsler Preschool and Primary Scale of Intelligence (Wechsler, 2003). In this task, participants were required to assemble a set of two‐dimensional puzzles depicting cartoon images of objects (e.g., bird, clock, car, fish). To date, very few studies of children's theory of mind (e.g., Low, 2010; Hughes, 1998) and, to our knowledge, no cross‐cultural studies of children's theory of mind have incorporated measures of nonverbal ability. We chose the object assembly task over the matrix reasoning task because it was suitable for use with 3‐ and 4‐year‐old children. In contrast, the matrix reasoning task can only be used with children aged over 4 years (Wechsler, 2003). The object assembly task is strongly correlated with performance IQ in 3‐ and 4‐year‐old children (.82 < *r* < .85) and shows acceptable 1‐month test–retest reliability (.74 < *r* < .76) in this age group (Wechsler, 2003). The task began with two training trials in which the experimenter demonstrated how to use the puzzle pieces. For example, in one training trial, the experimenter showed the child how two pieces fit together to make a picture of a ball and then asked the child to make the same picture. In each subsequent trial, the experimenter presented the child with the pieces for the puzzle and stated, “These pieces make a(n) ‘X’. Put them together as fast as you can and tell me when you're finished.” The number of correct junctures in the first 90 s was then recorded. Children completed up to a maximum of 14 trials (including the training trials). The scores for each trial were summed together giving a total possible range of 0 to 37 points.

#### Parental Mind‐Mindedness

Consistent with previous studies of preschool‐aged children, we used a brief interview to obtain a representational measure of parental mind‐mindedness (Meins & Fernyhough, 2015). The interview question was based on the 5‐min speech sample (Daley, Sonuga‐Barke, & Thompson, 2003). Prior to the interview, we informed parents that there were no right or wrong answers. We then read aloud the following instructions:

“I'd like to hear your thoughts and feelings about (child's name), in your own words and without my interrupting with any questions or comments. When I ask you to begin, I'd like you to speak for 5 minutes, telling me what kind of a person (child's name) is and how the two of you get along together.”

Parents’ responses were recorded using a digital recording device and later transcribed verbatim.

We coded the transcripts using the scheme developed by Meins and Fernyhough (2015). Individual descriptions of the child were coded into one of four exhaustive categories: mental (i.e., comments referring to the child's mental life), behavioral (i.e., comments referring to the child's behavior or routines), physical (i.e., comments referring to the child's appearance), or general (i.e., vague or unclear comments about the child not fitting the other three categories). Given that English was a common language between the two research teams, we established interrater reliability by double coding a random selection of 25% (*N *=* *30) of the U.K. transcripts and 12.5% (*N *=* *15) of the transcripts from the Hong Kong sample (translated into English). These transcripts were anonymized so that the two raters were unaware of the children's false belief test scores. The interrater reliability for coding each comment was good, κ = .79, *p* < .001. Intraclass correlations (ICC) for the total number of comments in each category were all significant, all *p*s < .001, indicating good interrater reliability for the total number of mental (ICC = .92), behavioral (ICC = .91), physical (ICC = .83), and general (ICC = .83) descriptions identified by each rater.

## Results

### Analytic Approach

We used a latent variable approach in *Mplus* Version 7 (Muthèn & Muthèn, 2012) to analyse our data. Given that our data included measures with non normal distributions, we used a mean‐ and variance‐adjusted weighted least squares estimator (rather than a maximum likelihood estimator) in each of our models (Brown, 2006; Kline, 2011). For each model, we evaluated the adequacy of fit using Brown's (2006) four recommended criteria: a nonsignificant chi‐square test, comparative fit index (CFI) ≥ 0.90, Tucker–Lewis index (TLI) ≥ 0.90, and root mean square error of approximation (RMSEA) ≤ 0.08. Effect sizes for the various model parameters were interpreted in accordance with recommendations from Kline (2011): small standardized effect sizes ranged from .10 to .30, moderate effect sizes ranged from .30 to .50, and large effects were > .50.

### Descriptive Statistics

Table 1 shows the descriptive statistics for each of the key study measures in the sample as a whole and separately by each country. Before data analysis, we inspected each of the key variables for extreme outliers (cases ± 3 *SD* from the mean) in the United Kingdom and Hong Kong samples. There were three mental description outliers and one behavioral description outlier in the Hong Kong data set. We report the analyses including these cases. Table 2 shows the correlations between each of the main study measures.

**Table 1:** Descriptive Statistics

	Whole sample			United Kingdom			Hong Kong		
	*M*	*SD*	Range	*M*	*SD*	Range	*M*	*SD*	Range
Age (years)	3.96	0.51	3–4.99	3.92	0.53	3–4.95	3.99	0.50	3.09–4.99
Nonverbal IQ	17.53	7.93	2–35	16.85	8.21	2–35	18.21	7.62	2–32
Language ability	14.77	7.15	0–36	15.12	7.43	0–36	14.43	6.87	2–32
Child‐aged siblings	0.65	0.62	0–3	0.68	0.61	0–3	0.61	0.64	0–3
False belief: Change of Location 1	0.82	0.78	0–2	0.88	0.79	0–2	0.75	0.78	0–2
False belief: Change of Location 2	0.95	0.83	0–2	0.97	0.81	0–2	0.93	0.85	0–2
False belief: Unexpected contents	0.83	0.81	0–2	1.03	0.86	0–2	0.62	0.71	0–2
False belief: Unexpected identity	0.97	0.81	0–2	1.02	0.82	0–2	0.93	0.82	0–2
Parental mind‐mindedness	11.09	6.02	0–29	13.18	5.74	1–29	9.03	5.60	0–24
Parental nonmental attributes	29.75	12.76	3–95	33.93	11.04	10–74	25.64	13.04	3–95
Hours/week in nursery	22.75	12.13	0–51	18.85	11.02	0–45	26.63	11.97	12–51

**Table 2:** Relations Between Key Study Variables in the United Kingdom (Above) and Hong Kong (Below)

	1	2	3	4	5	6	7	8	9	10	11	12	13	14
1. Age	—	.04	−.10	.50**	.53**	.05	.42**	.45**	.46**	.24**	.58**	−.03	−.03	.40**
2. Gender (F = 0, M = 1)	.21*	—	.11	.08	.01	.17+	−.02	−.06	−.16+	.02	−.08	−.15	−.02	.11
3. Parental education	−.03	−.08	—	.14	.05	−.01	−.05	.09	.01	−.06	.02	−.09	.08	.28**
4. Nonverbal ability	.51**	.21*	−.05	—	.43**	.12	.23*	.35**	.31**	.17+	.40**	.01	.10	.29**
5. Language ability	.51**	.14	−.01	.27**	—	−.08	.33**	.50**	.38**	.25**	.55**	.08	.09	.24**
6. Child‐aged siblings	−.05	.12	−.09	−.02	−.01	—	.03	.03	−.09	−.01	.001	−.05	−.01	−.08
7. False belief: CoL 1	.31**	.05	.15	.21*	.29**	−.03	—	.79**	.25**	.32**	—	.07	−.05	.07
8. False belief: CoL 2	.44**	.04	.13	.15	.39**	.06	.60**	—	.30**	.24**	—	.10	.04	.14
9. False belief: Contents	.04	.15	.10	−.04	.09	−.07	.34**	.22*	—	.47**	—	.20*	−.10	.09
10. False belief: Identity	.02	.07	−.18*	−.08	.04	−.01	.28**	.23*	.33**	—	—	.17+	.02	−.02
11. False belief latent factor	.48**	.10	.18*	.21*	.46**	.002	—	—	—	—	—	.19+	.16	.12
12. Parental mind‐mindedness	.14	−.04	.08	.09	.08	.04	.18*	.25*	.15	−.02	.31**	—	.31**	−.14
13. Nonmental attributes	.11	−.05	.07	.18*	.01	.01	.10	.09	.16+	.04	.16	.43**	—	.08
14. Hours/week at nursery	−.19*	.04	−.06	−.16+	.05	.05	−.06	.09	−.04	−.09	.02	−.04	−.10	—

Note.

Intercorrelations between the false belief task scores are tetrachoric correlations. Correlations involving the false belief latent factor were calculated using mean‐ and variance‐weighted least square estimation in *Mplus*. CoL = change of location.

+*p* < .10. *p < .05. **p < .01.

### Children's False Belief Understanding in the United Kingdom and Hong Kong

Our first aim was to compare children from the United Kingdom with children from Hong Kong in terms of false belief understanding. Consistent with previous studies (e.g., Hughes et al., 2014), the four false belief task indicators showed moderate tetrachoric correlations (see Table 2). Building on previously published studies (e.g., Hughes et al., 2014), we used CFA to examine the fit of a single latent factor for measuring individual differences in false belief understanding. That is, we hypothesized that each of the four categorical false belief task indicators would load onto a single latent factor. We scaled the metric of the latent factor by constraining the loading of the marker indicator (i.e., Change of Location 1) to 1. Given the similarity between the stories and questions used in the two change of location tasks, we specified a correlation between these two items. In doing so, we hoped to distinguish between task‐specific variance and variance due to false belief understanding (e.g., Brown, 2006). This single latent factor solution provided an excellent fit to the data, χ^2^(1) = 0.35, *p* = .56, CFI = 1, TLI = 1, RMSEA = 0.01. Each of the false belief task items loaded significantly onto the false belief understanding latent factor with standardized loadings ranging from .47 to .78, all *p*s < .001. Next, we evaluated the reliability of this latent factor by specifying a graded item response theory model using robust maximum likelihood estimation to estimate the precision of the false belief understanding task battery at different levels of the underlying latent factor (Embretson & Reise, 2000; Muthèn & Muthèn, 2012). Findings revealed that the false belief battery was most reliable when testing participants performing between 1 *SD* above and below the mean. For participants with average levels of false belief understanding, the reliability coefficient was .84. For those with lower than average ability (−1 *SD*) and higher than average ability (+1 *SD*), the reliability coefficients were .67 and .82, respectively.

To test the validity of the false belief understanding latent factor, we specified a second model in which we regressed each of the four false belief task indicators onto language scores. Once again, the model provided an excellent fit to the data, χ^2^(1) = 0.16, *p* = .69, CFI = 1, TLI = 1, RMSEA= 0.01. Supporting the validity of the false belief understanding latent factor, each of the four task indicators continued to load significantly onto the single latent factor (standardized loadings ranged from .31 to .69, all *p*s < .001). That is, language ability and shared method effects did not explain the relations between these task indicators.

Next, we tested the assumption of measurement invariance of the above single latent factor solution across the U.K. and Hong Kong samples using multiple groups CFA (Brown, 2006). To this end, we constrained the factor structure, item loadings, thresholds, correlated residuals, and latent factor variances to be equal across both countries. This model provided a good fit to the data, χ^2^(1) = 16.27, *p* = .09, CFI = 0.99, TLI = 0.99, RMSEA = 0.07, indicating that there was no evidence of bias favoring one group over the other. That is, the test items exhibited equivalent relations with the false belief latent factor in both countries. Next, we constrained the latent factor means to be equal across both groups in order to assess group differences in performance between children from the United Kingdom and Hong Kong. This constraint produced a significant decrease in model fit, Δχ^2^(1) = 8.74, *p* = .003, indicating a significant group difference. Specifically, as hypothesized, the children from the United Kingdom significantly out performed their Hong Kong counterparts on the false belief understanding latent factor, Cohen's *d *=* *0.47, *p* < .01, despite there being no significant differences between the two groups with respect to age, gender, verbal and nonverbal ability, and number of siblings.

### Parental Mind‐Mindedness in the United Kingdom and Hong Kong

Our second aim was to examine parental mind‐mindedness in the United Kingdom and Hong Kong. Inspection of the descriptive statistics showed that the ratio of mental to “nonmental” (i.e., behavioral, general, or physical) descriptions was broadly similar in both samples. Rather than using proportional scores, we examined the overall total number of descriptions made by parents during the interview. We chose to use this approach because the creation of proportion scores might artificially inflate or attenuate a parent's ranking. For example, if one parent were to give just one mental description of their child and two nonmental descriptions during the 5 min of the task and another parent were to give 20 mental descriptions and 40 nonmental descriptions, these parents would achieve the same “mental description” score. We therefore not only used raw scores but also included the total number of nonmental descriptions as a control measure of parental verbosity in our analyses (Meins & Fernyhough, 2015). Note that even when the total number of nonmental parental descriptions was included as a covariate in a between‐participants analysis of covariance, the British parents gave significantly more mental descriptions of their children than the Hong Kong parents, *F*(1, 238) = 6.45, *p* = .012, partial η^2^ = 0.03.

### Does Parental Mind‐Mindedness Explain Cultural Differences in False Belief Understanding?

**Figure 1:**
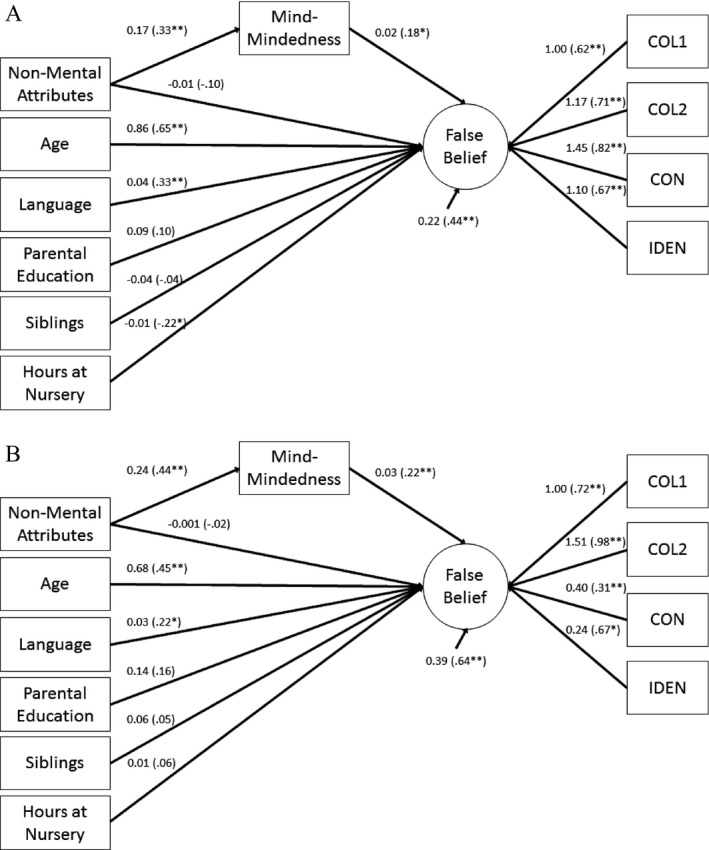
Predictors of individual differences in false belief understanding in the United Kingdom (A) and Hong Kong (B) samples.*Note*. *p < .05. **p < .01. Paths depict unstandardized estimates. Standardized estimates are shown in parentheses.

Our third and final aim was to examine whether the observed cultural differences in parental mind‐mindedness might account for the mean difference in false belief understanding between children from the United Kingdom and Hong Kong. Prior to examining cross‐cultural differences, we first examined the relations between parental mind‐mindedness and false belief understanding separately in each country. To this end, we examined the unique effect of parental mind‐mindedness on false belief latent factor scores by regressing children's performance on the latent factor onto age, gender, language ability, SES, family size, number of hours spent at nursery, and parental mind‐mindedness. We also entered the number of nonmental attributes provided by parents to control for overall parental verbosity. The results of these models are presented in Figures 1A and 1B. The model provided an excellent fit to the United Kingdom data, χ^2^(27) = 23.65, *p* = .65, CFI = 1.00, TLI = 1.00, RMSEA = 0. The model accounted for 66% of the variance in scores on the false belief latent factor. The model also provided an excellent fit to the Hong Kong data, χ^2^(27) = 31.11, *p* = .27, CFI = 0.97, TLI = 0.95, RMSEA = 0.04. This model accounted for 44% of the variance in scores on the false belief latent factor. Notably, parental mind‐mindedness was a significant (but weak) predictor of false belief understanding in both the United Kingdom and Hong Kong (see Figures 1A and 1B).

Using the analytic approach proposed by Preacher and Hayes (2004), we specified a model to examine whether parental mind‐mindedness mediated the effect of country (Hong Kong = 0, United Kingdom = 1) on children's false‐belief task performance. To control for potential confounds, we regressed the false‐belief latent factor onto children's age, language ability, number of child‐aged siblings, total number of hours per week spent at nursery, and parental SES. To account for differences in parental verbosity during the speech sample task, we also entered the total number of nonmental comments into the model as a predictor of both false belief understanding and parental mind‐mindedness.

**Figure 2:**
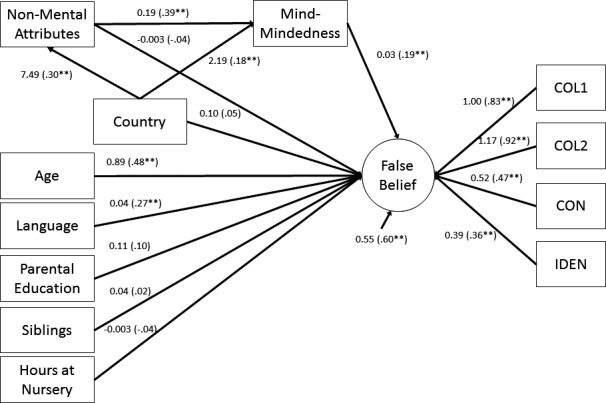
Mind‐mindedness mediates the link between country and false belief understanding.*Note*. **p < .01. Paths depict unstandardized estimates. Standardized estimates are shown in parentheses.

The model provided a good fit to the data, χ^2^(35) = 41.02, *p* = .22, CFI = 0.99, TLI = 0.98, RMSEA = 0.03. Figure 2 shows the unstandardized and standardized parameter estimates for this model, which accounted for 40% of the variance in scores on the false belief latent factor. Country exerted direct effects on both parental use of nonmental attributes and parental mind‐mindedness. As hypothesized, parental mind‐mindedness fully mediated the effect of country on children's false belief understanding, *B *=* *0.07, *SE* = 0.035, *Z *=* *1.97, *p* = .05, 95% CI [0.01, 0.12]. In contrast, nonmental descriptions did not mediate the relation between country and children's false belief understanding, *B *=* *−0.03, *SE* = 0.038, *Z *=* *−0.65, *p* = .51, 95% CI [−0.09, 0.04]. In addition, there was a significant indirect effect of country on false belief understanding via nonmental attributes and parental mind‐mindedness, *B *=* *0.04, *SE* = 0.019, *Z *=* *2.22, *p* = .03, 95% CI [0.01, 0.08]. That is, country predicted parents’ overall use of nonmental attributes, which in turn predicted parental mind‐mindedness and children's false belief understanding. Together, these results show that parental mind‐mindedness explained the observed differences between the United Kingdom and Hong Kong in children's theory of mind.

## Discussion

This cross‐cultural study of 241 parent–child dyads living in Hong Kong and the United Kingdom was motivated by meta‐analytic evidence for a puzzling delay in false belief understanding among preschoolers in Hong Kong relative to children in both mainland China and North America (Liu et al., 2008). It is the first study to include a direct comparison of theory‐of‐mind task performance in Hong Kong preschoolers and their Western counterparts. Using tests of measurement invariance, our findings confirmed the predicted delay among preschoolers in Hong Kong. This is also the first published study to examine parental mind‐mindedness in an Asian sample. Indeed, as noted elsewhere (e.g., Lewis, Huang, & Rooksby, 2006), measures of children's social environments have rarely been included in cross‐cultural studies in this field. Our findings highlight the universality of parental mind‐mindedness as a predictor of children's theory‐of‐mind performance, as there were significant associations between these two constructs in both cultures. This universal association is striking because, on average, parents in Hong Kong were much less likely than their British counterparts to describe their children using mental attributes. Importantly, our mediation model showed that the group difference in parental mind‐mindedness explained the group difference in children's theory‐of‐mind task performance. This is a critical step for cross‐cultural research in this field which has been largely restricted to reports of similarities or contrasts in child performance with almost no direct testing of the social factors that may underpin any group differences.

### Children's False Belief Understanding in the United Kingdom and Hong Kong

Our first set of findings add to a growing body of literature demonstrating marked differences between cultures in children's ability to reason about beliefs. It is interesting to note that although significant, the effect size (Cohen's *d*) for the difference between our two matched groups of participants was substantially smaller than those reported by Liu et al. (2008). Effect sizes for the differences between Hong Kong and North American children's false belief task performance were 0.92 (for Hong Kong vs. U.S.) and 1.42 (for Hong Kong vs. Canada; Liu et al., 2008). One obvious reason for this difference is that the previously reported meta‐analytic findings relied on an indirect comparison between Hong Kong children and children in North America. That is, the studies in the meta‐analysis were single culture studies. Several methodological shortcomings may also constrain the conclusions that can be drawn from existing cross‐cultural findings. These include a heavy reliance on simple pass/fail measures (which are not sensitive to individual differences) and a widespread failure to control for key background variables such as verbal ability or to test for measurement invariance (Hughes et al., 2014). Despite addressing these potential methodological confounds and testing the measurement invariance of our battery of false‐belief tasks, our study still revealed a substantial difference in children's theory‐of‐mind task performance. Thus, the observed differences in theory of mind between children in the United Kingdom and Hong Kong are unlikely to be due solely to methodological factors.

### Parental Mind‐Mindedness in the United Kingdom and Hong Kong

Our study findings showed that, compared with parents in the United Kingdom, parents in Hong Kong offered fewer descriptions of their children's attributes in general (and mental attributes in particular) than did parents in the United Kingdom. Note that the parental speech samples were gathered in equivalent settings (a university laboratory room) across sites using precisely the same instructions and protocol, such that the contrasting number of child‐focused descriptions did not reflect a methodological artifact of contextual cues.

How might contrasts in parental mind‐mindedness be explained? One possibility is that the observed cross‐cultural differences in parental mind‐mindedness (and indeed children's false belief understanding) could be explained by sharp differences in parental education between the two samples. Indeed, meta‐analytic results show that individual differences in children's false belief understanding are moderately correlated with parental SES (Devine & Hughes, 2018). Against this view, however, parental mind‐mindedness was unrelated to parental education in either sample; this lack of association is consistent with previous findings from studies involving samples with lower average levels of education (e.g., Meins et al., 2003). Moreover, in contrast the parental education was statistically controlled for in each of our models. Thus, our current findings do not support the view that the cross‐cultural contrast in mind‐mindedness could be explained by differences in parental education. However, matching samples for parental education is an obvious goal for future cross‐cultural studies of theory of mind.

In a thoughtful discussion of the social contexts in which mentalizing skills are likely to prove useful, Ames et al. (2001) noted that situations involving strong social norms are likely to place relatively few demands on an individual's ability to read others’ minds. From this perspective, it is worth noting that the very high value attached to filial piety within Asian cultures means that children are expected to conform to social norms regardless of their own views. In other words, echoing distinctions between learned social conventions and complex interactional social skills (Frith, Happé, & Siddons, 1994), parental socialization of children in particular cultures may not require fostering children's social understanding. Support for this view comes from a study that directly compared the socialization goals of parents living in the United Kingdom and in Hong Kong. Specifically, Pearson and Rao (2003) found that Hong Kong parents were, on average, much more likely than their United Kingdom counterparts to place a high value on filial piety. Moreover, in both countries, an emphasis on filial piety was associated with authoritarian parenting, whereas an emphasis on socioemotional skills was associated with authoritative parenting. As noted in the Introduction, parental mind‐mindedness is positively related to sensitive parenting and therefore might be inversely related to authoritarian parenting, such that the results from Pearson and Rao's (2003) study of socialization goals might provide a mirror image of the findings from the present study. In other words, a parental emphasis on individual thoughts and interests may well hinder rather than help achieve socialization goals if, for example, those goals are focused on fostering filial piety. Exploring the links between mind‐mindedness and parenting style is a promising avenue for future research.

The current study makes a clear contribution to the literature in demonstrating cross‐cultural contrasts in both children's false belief understanding and parental mind‐mindedness and cultural invariance in the association between these two measures. That said, by including just two sites, it is difficult to avoid conclusions that simply perpetuate old stereotypes regarding contrasts between East and West (Wang & Chang, 2012). The substantial differences in parenting styles between families living in China and in Hong Kong that have been reported in several large‐scale studies (e.g., Berndt et al., 1993) demonstrate the multiplicity of cultures encompassed by the terms “East.” Future cross‐cultural studies in this field should therefore include at least three samples to achieve a more fine‐grained picture of the processes underpinning social influences on children's acquisition of a theory of mind. For example, a recent study of school‐aged children living in the United Kingdom or Hong Kong has shown that the pattern of between‐country differences in theory of mind and executive function is dependent on whether the Hong Kong sample is recruited from local or international schools (Wang, Devine, Wong, & Hughes, 2016). Specifically, compared with their United Kingdom counterparts, both Hong Kong samples showed superior executive function, and only the Hong Kong children attending local schools lagged behind their United Kingdom peers on tests of theory of mind. Beyond highlighting the importance of comparing more than two samples, this differentiated pattern of results underscores the need to adopt a similar multifaceted approach in future work.

### Parental Mind‐Mindedness and False Belief Understanding

Our third set of findings related to the links between parental mind‐mindedness and children's false belief understanding. Interestingly the links between these two constructs were significant and similar in magnitude across countries despite the group differences in mean levels of both parental mind‐mindedness and children's theory‐of‐mind task performance. The universality of associations between mind‐mindedness and children's theory of mind strengthens accounts of familial influences on theory of mind that focus on the expert tuition provided by mind‐minded parents (e.g., Heyes & Frith, 2014). Moreover, our findings demonstrated that when group differences in parental mind‐mindedness were taken into account, the cross‐cultural contrast in children's theory‐of‐mind task performance attenuated. This point underscores the independence of group differences and within‐group associations. That is, even in a culture in which mind‐mindedness is not a particularly prominent feature of caregiving, within‐group variation can still have meaningful correlations. Of course, the cross‐sectional nature of our data set constrains any conclusions about the developmental role of parental mind‐mindedness in children's false belief understanding. That said our findings provide an exciting starting point for cross‐cultural investigations of familial influences on children's theory of mind.

Future studies might examine potential contrasts in the nature and relative salience of predictors of individual differences in theory of mind for children from different cultures. Although the variables included in the current study explained an impressive 64% of the variation in performance on tests of theory of mind among preschoolers in the United Kingdom, the same measures explained just 44% of the corresponding variation among Hong Kong preschoolers. In addition, hours spent in nursery were inversely related to theory of mind in British preschoolers but were unrelated to theory of mind in Hong Kong preschoolers. Together, these findings indicate the need to widen the net in order to identify other variables that might contribute to individual differences in theory of mind in non‐Western children. In a recent meta‐analysis on family correlates of theory of mind, Devine and Hughes (2018) identified four factors that have been widely studied: SES, family size, parental mind‐mindedness, and mental‐state talk. Interestingly, very few of the included studies in this review included hours in nonparental care as a predictor variable. Moreover, the interplay and overlap between these family correlates of false belief task performance also remain largely unexplored. Our study highlights the need for further investigation in this area.

### Conclusions

The current study provides the first examination of the links between parental mind‐mindedness and children's theory of mind in two cultures. Our results indicate cross‐cultural differences in both preschool children's understanding of false beliefs and in parental mind‐mindedness. Consistent with sociocultural accounts of children's theory‐of‐mind development, we propose that the observed cross‐cultural differences in children's mental‐state reasoning are unlikely to be due solely to methodological artifacts. Instead, we argue that the results support the conclusion that cross‐cultural disparities in children's ability to reason about false beliefs probably reflect contrasts in aspects of family life related to parental mind‐mindedness.

## References

[cdev12746-bib-0001] Adams, A. , & Gathercole, S. E. (1996). Phonological working memory and spoken language development in young children. Quarterly Journal of Experimental Psychology, 49A, 216–233. doi:10.1080/713755610

[cdev12746-bib-0002] Adesope, O. O. , Lavin, T. , Thompson, T. , & Ungerleider, C. (2010). A systematic review and meta‐analysis of the cognitive correlates of bilingualism. Review of Educational Research, 80, 207–245. doi:10.3102/0034654310368803

[cdev12746-bib-0003] Ames, D. R. , Knowles, E. D. , Morris, M. W. , Kalish, C. W. , Rosati, A. D. , & Gopnik, A. (2001). The social folk theorist: Insights from social and cultural psychology on the contents and contexts of folk theorizing In Malle B., Moses L., & Baldwin D. (Eds.), Intentions and intentionality: Foundations of social cognition (pp. 307–330). Cambridge, MA: MIT Press.

[cdev12746-bib-0004] Baron‐Cohen, S. , Leslie, A. M. , & Frith, U. (1985). Does the autistic child have a “theory of mind”? Cognition, 21, 37–46. doi:10.1016/0010-0277(85)90022-8 2934210

[cdev12746-bib-0005] Berndt, T. J. , Cheung, P. C. , Lau, S. , Hau, K. , & Lew, W. J. F. (1993). Perceptions of parenting in Mainland China, Taiwan and Hong Kong: Sex differences and societal differences. Developmental Psychology, 29, 156–164. doi:10.1037/0012-1649.29.1.156

[cdev12746-bib-0006] Bornstein, M. H. , Putnick, D. L. , Cote, L. R. , Haynes, O. M. , & Suwalsky, J. T. D. (2015). Mother‐infant contingent vocalisations in 11 countries. Psychological Science, 26, 1272–1284. doi:10.1177/0956797615586796 26133571 PMC4529355

[cdev12746-bib-0007] Bornstein, M. H. , Tal, J. , Rahn, C. , Galperin, C. , Lamour, M. , Ogino, M. , … Tamis‐LeMonda, C. (1992). Functional analysis of the contents of maternal speech to infants of 5 and 13 months in four cultures: Argentina, France, Japan and the United States. Developmental Psychology, 28, 593–603. doi:10.1037/0012-1649.28.4.593

[cdev12746-bib-0008] Brown, T. A. (2006). Confirmatory factor analysis for applied research. London, UK: Guilford.

[cdev12746-bib-0009] Byrne, B. M. , & Campbell, T. L. (1999). Cross‐cultural comparisons and the presumption of equivalent measurement and theoretical structure: A look beneath the surface. Journal of Cross‐Cultural Psychology, 30, 555–574. doi:10.1177/0022022199030005001

[cdev12746-bib-0010] Callaghan, T. , Rochat, P. , Lillard, A. , Claux, M. L. , Odden, H. , Itakura, S. , … & Singh, S. (2005). Synchrony in the onset of mental‐state reasoning: Evidence from five cultures. Psychological Science, 16, 378–84. doi:10.1111/j.0956-7976.2005.01544.x 15869697

[cdev12746-bib-0011] Carruthers, P. (2013). Mindreading in infancy. Mind & Language, 28, 141–172. doi:10.1111/mila.12014

[cdev12746-bib-0012] Chen, F. F. (2008). What happens if we compare chopsticks with forks? The impact of making inappropriate comparisons in cross‐cultural research. Journal of Personality and Social Psychology, 95, 1005–1018. doi:10.1037/a0013193 18954190

[cdev12746-bib-0013] Cutting, A. L. , & Dunn, J. (1999). Theory of mind, emotion understanding, language, and family background: Individual differences and interrelations. Child Development, 70, 853–865. doi:10.1111/1467-8624.00061 10446724

[cdev12746-bib-0014] Daley, D. , Sonuga‐Barke, E. J. S. , & Thompson, M. (2003). Assessing expressed emotion in mothers of preschool AD/HD children: Psychometric properties of a modified speech sample. British Journal of Clinical Psychology, 42, 53–67. doi:10.1348/014466503762842011 12675979

[cdev12746-bib-0015] Devine, R.T. , & Hughes, C. (2018). Family correlates of false belief understanding in early childhood: A meta‐analysis. Child Development, 89, 971–987. doi: 10.1111/cdev.12682 27883189

[cdev12746-bib-0016] Douglas, S. P. , & Craig, C. S. (2007). Collaborative and iterative: An alternative approach to back translation. Journal of International Marketing, 15, 30–43. doi:10.1509/jimk.15.1.030

[cdev12746-bib-0017] Downey, D. B. (2001). Number of siblings and intellectual development: The resource dilution explanation. American Psychologist, 56, 497–504. doi:10.1037/0003-066X.56.6-7.497 11413873

[cdev12746-bib-0200] Embretson, S. E. , & Reise, S. P. (2000). Item response theory of psychologists. London, UK: Lawrence Erlbaum Associates.

[cdev12746-bib-0018] Frith, U. , Happé, F. , & Siddons, F. (1994). Autism and theory of mind in everyday life. Social Development, 3, 108–124. doi:10.1111/j.1467-9507.1994.tb00031.x

[cdev12746-bib-0019] Gopnik, A. , & Astington, J. W. (1988). Children's understanding of representational change and its relation to the understanding of false belief and the appearance‐reality distinction. Child Development, 59, 26–37. doi:10.2307/1130386 3342716

[cdev12746-bib-0020] Heyes, C. M. , & Frith, C. D. (2014). The cultural evolution of mind reading. Science, 344, 1357. doi:10.1126/science.1243091 24948740

[cdev12746-bib-0021] Hong Kong Education Bureau . (n.d.). Distribution of educational attainment of population aged 15 and over. Retrieved from: http://www.edb.gov.hk/en/about-edb/publications-stat/figures/educational-attainment.html

[cdev12746-bib-0022] Hughes, C. (1998). Finding your marbles: Does pre‐schoolers’ strategic behavior predict later understanding of mind? Developmental Psychology, 34, 1326–1339. doi:10.1037/0012-1649.34.6.1326 9823515

[cdev12746-bib-0023] Hughes, C. , & Devine, R. T. (2015). A social perspective on theory of mind In Lamb M. E. (Ed.), Handbook of child psychology and developmental science (Volume III): Socioemotional processes (pp. 564–609). Hoboken, NJ: Wiley.

[cdev12746-bib-0024] Hughes, C. , Devine, R. T. , Ensor, R. , Koyasu, M. , Mizokawa, A. , & Lecce, S. (2014). Lost in translation? Comparing British, Japanese and Italian children's theory of mind performance. Child Development Research, 893492. doi:10.1155/2014/893492

[cdev12746-bib-0025] Kline, R. B. (2011). Principles and practice of structural equation modeling (3rd ed.). London, UK: Guilford.

[cdev12746-bib-0026] Kovas, Y. , Hayiou‐Thomas, M. E. , Oliver, B. , Dale, P. S. , Bishop, D. V. M. , & Plomin, R. (2005). Genetic influences in different aspects of language development: The etiology of language skills in 4.5‐year‐old twins. Child Development, 76, 632–651. doi:10.1111/j.1467-8624.2005.00868.x 15892783

[cdev12746-bib-0027] Lai, A. C. , Zhang, Z. X. , & Wang, W. Z. (2000). Maternal child‐rearing practices in Hong Kong and Beijing Chinese families: A comparative study. International Journal of Psychology, 35, 60–66. doi:10.1080/002075900399529

[cdev12746-bib-0028] Laranjo, J. , Bernier, A. , Meins, E. , & Carlson, S. (2014). The roles of maternal mind‐mindedness and infant security of attachment in pre‐schoolers’ understanding of visual perspectives and false belief. Journal of Experimental Child Psychology, 125, 48–62. doi:10.1016/j.jecp.2014.02.005 24814206

[cdev12746-bib-0029] Lecce, S. , & Hughes, C. (2010). “The Italian job?”: Comparing theory of mind performance in British and Italian children. British Journal of Developmental Psychology, 28, 747–766. doi:10.1348/026151009X479006 21121465

[cdev12746-bib-0030] Lewis, C. , Huang, Z. , & Rooksby, M. (2006). Chinese pre‐schoolers’ false belief understanding: Is social knowledge underpinned by parental styles, social interactions or executive functions? Psychologia: An International Journal of Psychology in the Orient, 49, 252–266. doi:10.2117/psysoc.2006.252

[cdev12746-bib-0031] Lillard, A. (1998). Ethnopsychologies: Cultural variations in theories of mind. Psychological Bulletin, 123, 3–32. doi:10.1037/0033-2909.123.1.3 9461850

[cdev12746-bib-0032] Liu, D. , Wellman, H. M. , Tardif, T. , & Sabbagh, M. A. (2008). Theory of mind development in Chinese children: A meta‐analysis of false‐belief understanding across cultures and languages. Developmental Psychology, 44, 523–531. doi:10.1037/0012-1649.44.2.523 18331141

[cdev12746-bib-0501] Low, J. (2010). Preschoolers' implicit and explicit false belief understanding: Relations with complex syntactical mastery. Child Development, 81, 597–615. 20438463 10.1111/j.1467-8624.2009.01418.x

[cdev12746-bib-0033] Lundy, B. L. (2013). Paternal and maternal mind‐mindedness and pre‐schoolers’ theory of mind: The mediating role of interactional attunement. Social Development, 22, 58–74. doi:10.1111/sode.12009

[cdev12746-bib-0034] Meins, E. , & Fernyhough, C. (2015). Mind‐mindedness coding manual (2nd ed.). York, UK: University of York.

[cdev12746-bib-0035] Meins, E. , Fernyhough, C. , Arnott, B. , Leekam, S. R. , & DeRosnay, M. (2013). Mind‐mindedness and theory of mind: Mediating roles of language and perspectival symbolic play. Child Development, 84, 1777–1790. doi:10.1111/cdev.12061 23432622

[cdev12746-bib-0036] Meins, E. , Fernyhough, C. , DeRosnay, M. , Arnott, B. , Leekam, S. R. , & Turner, M. (2012). Mind‐mindedness as a multi‐dimensional construct: Appropriate and nonattuned mind‐related comments independently predict infant‐mother attachment in a socially diverse sample. Infancy, 17, 393–415. doi:10.1111/j.1532-7078.2011.00087.x 32693485

[cdev12746-bib-0037] Meins, E. , Fernyhough, C. , Fradley, E. , & Tuckey, M. (2001). Rethinking maternal sensitivity: Mothers’ comments on infants’ mental processes predict security of attachment at 12 months. Journal of Child Psychology and Psychiatry, 42, 637–648. doi:10.1111/1469-7610.00759 11464968

[cdev12746-bib-0038] Meins, E. , Fernyhough, C. , Russell, J. , & Clark‐Carter, D. (1998). Security of attachment as a predictor of symbolic and mentalizing abilities: A longitudinal study. Social Development, 7, 1–24. doi:10.1111/1467-9507.00047

[cdev12746-bib-0039] Meins, E. , Fernyhough, C. , Wainwright, R. , Clark‐Carter, D. , Das Gupta, M. , Fradley, E. , & Tuckey, M. (2003). Pathways to understanding mind: Construct validity and predictive validity of parental mind‐mindedness. Child Development, 74, 1194–1211. doi:10.1111/1467-8624.00601 12938713

[cdev12746-bib-0040] Meins, E. , Fernyhough, C. , Wainwright, R. , Das Gupta, M. , Fradley, E. , & Tuckey, M. (2002). Maternal mind‐mindedness and attachment security as predictors of theory of mind understanding. Child Development, 73, 1715–1726. doi:10.1111/1467-8624.00501 12487489

[cdev12746-bib-0041] Milligan, K. , Astington, J. W. , & Dack, L. A. (2007). Language and theory of mind: Meta‐analysis of the relation between language ability and false‐belief understanding. Child Development, 78, 622–646. doi:10.1111/j.1467-8624.2007.01018.x 17381794

[cdev12746-bib-0042] Moerbeek, K. (1994). Can't sleep. London, UK: Golden Books.

[cdev12746-bib-0043] Muthèn, L. K. , & Muthèn, B. O. (2012). Mplus: Statistical analysis with latent variables. User's guide (7th ed.). Los Angeles, CA: Author.

[cdev12746-bib-0044] Nisbett, R. E. , & Masuda, T. (2003). Culture and point of view. Proceedings of the National Academy of Sciences of the United States of America, 100, 11163–11170. doi:10.1073/pnas.1934527100 12960375 PMC196945

[cdev12746-bib-0045] Office of National Statistics (UK) . (2012). Highest level of qualification (Cambridge). Retrieved from http://www.ukcensusdata.com/cambridge-e07000008#sthash.|IXIIwmm.aPnhUGeP.dpbs

[cdev12746-bib-0046] Pearson, E. , & Rao, N. (2003). Socialization goals, parenting practices, and peer competence in Chinese and English pre‐schoolers. Early Child Development and Care, 173, 131–146. doi:10.1080/0300443022000022486

[cdev12746-bib-0047] Perner, J. , Mauer, M. C. , & Hildenbrand, M. (2011). Identity: Key to children's understanding of belief. Science, 333, 474–477. doi:10.1126/science.1201216 21778403

[cdev12746-bib-0048] Peterson, C. (2000). Kindred spirits: Influence of siblings’ perspectives on theory of mind. Cognitive Development, 15, 435–455. doi:10.1016/S0885-2014(01)00040-5

[cdev12746-bib-0049] Posada, G. , Trumbell, J. , Noblega, M. , Plata, S. , Pena, P. , Carbonell, O. A. , & Lu, T. (2016). Maternal sensitivity and child secure base use in early childhood: Studies in different contexts. Child Development, 87, 297–311. doi:10.1111/cdev.12454 26525825

[cdev12746-bib-0050] Preacher, K. J. , & Hayes, A. F. (2004). SPSS and SAS procedures for estimating indirect effects in simple mediation models. Behavior Research Methods, 36, 717–731. doi:10.3758/BF03206553 15641418

[cdev12746-bib-0051] Renfrew, C. E. (1997). The Renfrew Language Scales Bus Story Test: A test of narrative speech. Bicester, UK: Speechmark.

[cdev12746-bib-0052] Sabbagh, M. A. , Xu, F. , Carlson, S. M. , Moses, L. J. , & Lee, K. (2006). The development of executive functioning and theory of mind. A comparison of Chinese and U.S. pre‐schoolers. Psychological Science, 17, 74–81. doi:10.1111/j.1467-9280.2005.01667.x 16371147 PMC2567057

[cdev12746-bib-0053] Scholl, B. J. , & Leslie, A. M. (2001). Minds, modules and meta‐analysis. Child Development, 72, 696–701. doi:10.1111/1467-8624.00308 11405575

[cdev12746-bib-0054] Stokes, S. F. , & Wong, A. M. (1996). Validation of a Cantonese version of the Preschool Language Assessment Instrument. Asia Pacific Journal of Speech, Language and Hearing, 1, 75–90. doi:10.1179/136132896805577496

[cdev12746-bib-0055] Taumoepeau, M. (2015). From talk to thought: Strength of ethnic identity and caregiver mental state talk predict social understanding in pre‐schoolers. Journal of Cross‐Cultural Psychology, 46, 1169–1190. doi:10.1177/0022022115604393

[cdev12746-bib-0500] Taylor, M. , Cartwright, B. S. , & Bowden, T. (1991). Perspective taking and theory of mind: Do children predict interpretive diversity as a function of differences in observers' knowledge? Child Development, 62, 1334‐1351. doi:10.2307/1130810 1786719

[cdev12746-bib-0056] Vinden, P. G. (1999). Children's understanding of mind and emotion: A multi‐culture study. Cognition & Emotion, 13, 19–48. doi:10.1080/026999399379357

[cdev12746-bib-0057] Wang, Q. , & Chang, L. (2012). Parenting and child socialisation in contemporary China In Harris Bond M. (Ed.). Oxford handbook of Chinese psychology (pp. 53–68). Oxford, UK: Oxford University Press.

[cdev12746-bib-0058] Wang, Z. , Devine, R. T. , Wong, K. K. , & Hughes, C. (2016). Theory of mind and executive function during middle childhood across cultures. Journal of Experimental Child Psychology, 149, 6–22. doi:10.1016/j.jecp.2015.09.028 26592766

[cdev12746-bib-0059] Wang, Q. , Doan, S. N. , & Song, Q. (2010). Talking about internal states in mother‐child reminiscing influences children's self‐representations: A cross‐cultural study. Cognitive Development, 25, 380–393. doi:10.1016/j.cogdev.2010.08.007 21076662 PMC2976551

[cdev12746-bib-0060] Wechsler, D. (2003). Wechsler Preschool and Primary Scale of Intelligence (WIPPSI‐UK) (3rd ed.). London, UK: Harcourt Assessment.

[cdev12746-bib-0061] Wellman, H. M. , Cross, D. , & Watson, J. (2001). Meta‐analysis of theory‐of‐mind development: The truth about false belief. Child Development, 72, 655–684. doi:10.1111/1467-8624.00304 11405571

